# Human periodontitis-associated salivary microbiome affects the immune response of diabetic mice

**DOI:** 10.1080/20002297.2022.2107814

**Published:** 2022-08-04

**Authors:** Jinzhi He, Xin Shen, Di Fu, Yutao Yang, Kaixin Xiong, Lei Zhao, Huixu Xie, Georege Pelekos, Yan Li

**Affiliations:** a State Key Laboratory of Oral Diseases & National Clinical Research Center for Oral Diseases, West China Hospital of Stomatology, Chengdu, Sichuan, China; b Department of Cariology and Endodontics, West China Hospital of Stomatology, Sichuan University, Chengdu, Sichuan, China; c Faculty of Dentistry, The University of Hong Kong, Hong Kong, China

**Keywords:** Periodontitis, diabetes mellitus, microbiome, dysbiosis, immune cells, cytokines

## Abstract

**Background:**

The bidirectional association between periodontitis and diabetes mellitus has been well accepted; however, pathways connecting them remain unclear. Some oral bacteria are able to induce immunologic changes favoring insulin resistance individually. However, it is unclear if and how the systemic immune system responds to a disturbed oral microbial community in diabetic sufferers.

**Aim:**

This study aimed to investigate the impact of the human periodontitis-associated salivary microbiome on the splenic immune responses of diabetic mice.

**Methods:**

An *in vivo* diabetic animal model was established by feeding high fat food. After microbial depletion with quadruple antibiotic treatment, human saliva from healthy and periodontitis volunteers was transplanted into the mouth of these diabetic mice (N = 3), respectively.

**Results:**

Osteoclasts and expression levels of TNF-α and IL-1β were significantly increased in periodontal tissues of mice receiving periodontitis patients donated microbiome compared to these transplanted with healthy subjects donated microbiome. The proportion of monocyte (an innate immunocyte) decreased in mice receiving periodontitis patients donated microbiome. However, the abundance of an adaptive immunocyte Th17 was up-regulated. The IL17 production of ILC3 cells in human periodontitis-associated salivary microbiome recipient mice was significantly impaired.

**Conclusions:**

A disturbed oral microbiome imposes a stress on the splenic immune responses of diabetic mice.

## Introduction

Periodontitis is an inflammatory disease occurring in the tooth-supporting structures [1]. It is the major reason for tooth loss throughout the world [1]. Worse still, periodontitis has been implicated to be a risk factor for several systemic diseases, of which the most well known is diabetes mellitus (DM) [2]. DM is a group of metabolic disorders due to defects in insulin secretion and/or insulin action. It is characterized by chronic hyperglycemia with disturbances in carbohydrates, fat and protein metabolism [3]. Periodontitis adversely affects glycemic control in diabetic patients and aggravates the development of diabetic complications [4], while periodontal therapy results in a modest improvement of glycemic control in individuals with DM [5]. A bidirectional relationship between periodontitis and DM has been revealed by the fact that the risk for periodontitis increases 2 ~ 3 times in DM patients compared to people with normal glycemia [2]. Exploring the mechanisms underlying the two-way relationship between periodontitis and DM not only has important clinical implications for developing strategies to treat these two widespread diseases but also provides crucial clues to understand the vicious feed-forward loop between oral and systemic diseases.

It has been well accepted that initiation and propagation of periodontitis occur through a dysbiosis of the oral commensal microbial community [6]. A primary virulence of the oral microbiota leading to periodontal destruction is activating the host immune cascade [7,8]. At the same time, pivotal roles of the immune response have been well documented in the ethiopathogenicity of DM [9]. Interestingly, oral pathogens, such as *Porphyromonas gingivalis*, are able to mediate not only a local immune response within periodontal pockets but systemic immune response enhancing insulin resistance [10,11]. The above findings suggest that the oral bacteria-immune axis is the bridge connecting DM and periodontitis and that the dysbiotic oral microbiota in periodontitis might participate in the progression of DM by influencing the distally systemic immune response. Consistent with this hypothesis, pioneer studies have shown that oral pathogens are able to affect the systemic immune responsiveness involved in DM [10,11]. However, these studies were carried out with a mono-infection model and/or focused on the influence of a certain pathogen on specific immunocytes. Whereas it is a useful reductionist experimental strategy which renders the complexities of immune response–microbiota interactions more tractable, it sets aside the combinatorial effects of the microbiota within a complex community. It remains unclear how the innate and adaptive arms of the systemic immune systems respond to the disequilibrium of the entire oral microbial community in the scenario of DM.

Microbiota transplantation is the administration of a microbial community from a donor into a recipient. The best example showing the advantages of microbiota transplantation is fecal microbiota transplantation (FMT), which not only helps us acquire functional profiles of the microbiome but has been proven to be a very efficient therapeutic intervention [12]. Although the conception of oral microbiota transplantation (OMT) has been suggested, few oral transplantation studies have been reported [13–15]. In the present study, by combining a murine DM animal model, holistic OMT and multi-color flow cytometry, we investigated the influence of the oral microbial community disturbance on the systemic immune response (i.e. the splenic immune reaction) of mice with DM.

## Materials and methods

### Ethical approval

The present study was approved by the Institutional Review Board of the West China Hospital of Stomatology, Sichuan University (WCHSIRB-D-2017-069 and WCHSIRB-D-2017-035). Animals were handled according to the guidelines of the Institutional Authority for laboratory Animal Care at Sichuan University.

### Saliva collection

Sex- and age-matched periodontitis patients (N = 8) and healthy controls (N = 12) were recruited at the West China Hospital of Stomatology, Sichuan University. Inclusion criteria for periodontitis patients included (i) 20 ~ 65 years old, (ii) medically healthy, (iii) no previous periodontal treatment and antibiotics use within the past half-year, (iv) attachment loss >3 mm, (v) probe depth >6 mm. Periodontally healthy subjects had (i) no periodontal pockets, (ii) no clinical attachment loss, (iii) no alveolar bone absorption, and (iv) less than 15% of periodontal sites with bleeding on probing or redness. A writing consent was obtained before microbial sampling. All volunteers were asked to refrain from food and drink 1 h before saliva donation. Saliva was sampled in the morning (8:00 am~10:00 am). Approximately 5 mL of spontaneous, unstimulated whole saliva was expectorated into a sterile 50 mL cryogenic vial. Saliva was centrifuged at 500 g for 2 min to remove food debris. The saliva of healthy controls and periodontitis patients was equal volume pooled, respectively, labelled as periodontitis patient donated or healthy subject donated microbiome, aliquoted, and stored at −80°C until use.

### 16S rRNA sequencing

Aliquots of periodontitis patient-donated microbiome and healthy subject-donated microbiome were used for 16S rRNA sequencing. The DNA was extracted, quantified, and amplified with primer F338 and R806 targeting the V3-V4 region. The sequencing was done by Illumina HiSeq technology sequencing. The data were analyzed as described before [16,17]. Briefly, the sequences were clustered into operational taxonomic units (OTUs) at the 97% similarity level and were binned to phyla and genera using the Classifier at RDP-II against the Silva database. The relative abundance was calculated based on the proportion of reads.

### In vivo diabetic mouse model establishment

C57BL/6 mice (female, 8 weeks old, N = 3 per group) were housed in a specific pathogen-free facility. Animals were fed with high fat food (D12492, Research diets) to induce DM [18]. Fast plasma glucose levels were checked using an Accu-Chek Performa Glucometer. The oral glucose tolerance test (OGTT) was also carried out to double-check if the model was successfully established at the start point, after 2 and 4 months, respectively. For the OGTT, glucose was delivered via gavage, and blood glucose levels were measured at 30 min before injection and 30, 60, and 120 min after gavage. Meanwhile, the area under the curve (AUC) of the OGTT test was calculated as described before [19].

### Oral microbiota transplantation

**Figure 1.**
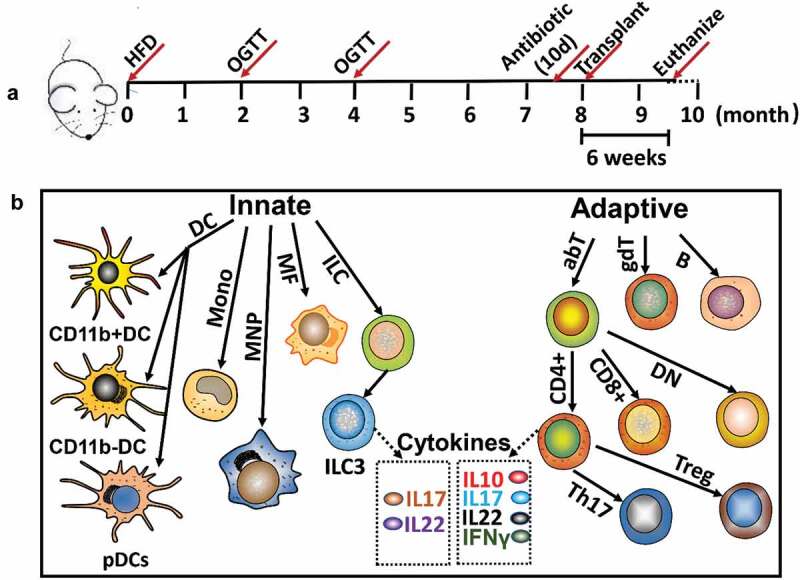
Experiment design. (a) Treatment regime; (b) Innate and adaptive immune cells and cytokines of spleen analyzed by flow cytometry. DC: dendritic cell; pDCs: plasmacytoid dendritic cell; Mono: monocyte; MNP: mononuclear phagocyte; MF: macrophage; ILC: innate lymphocyte cell; ILC3: innate lymphocyte cell type 3; abT: TCRαβ T cell; gdT: TCRγδ T cell; B: B cell; CD4+: CD4+ cell; CD8+: CD8+ cell; DN: CD4-CD8-T cell; Th17: Th17 T cell; Treg: Foxp3+ regulatory T cell.

To virtually deplete the oral microbiota, the animals were subjected to quadruple antibiotic treatment [20]. The antibiotics at defined concentrations (1 g/L Ampicillin, 1 g/L Neomycin, 1 g/L Metronidazole and 0.5 g/L Vancomycin) were added to the water, and the treatment lasted for 10 days. Animals were then randomly grouped into HSDM (healthy subject donated microbiome recipient mice) and PPDM group (periodontitis patient donated microbiome recipient mice). Pooled human saliva (200 uL) was transplanted into the mouse mouth, and the transplantation was done twice a week for 2 weeks. The animals were euthanized 6 weeks after oral microbiota transplantation. The regime for diabetic mouse model establishment and oral microbiota transplantation are given in Figure 1(a).

### Liver and kidney function tests

Blood was sampled by eyeball extirpating under terminal anesthesia. The serum was isolated, and the concentration of high-density lipoprotein (HDL-C), low-density lipoprotein (LDL-C), total cholesterol (TC) [21], triglyceride (TG), alanine aminotransferase (ALT), creatinine (CREA) and urea (UREA) were further measured by an automatic biochemical analyzer (AU680, Beckman, CA).

### microCT scanning of mandibles

Mandibles were dissected under a microscope and then fixed in 4% paraformaldehyde overnight at room temperature. Scanning was done with a micro-CT (μCT50; SCANCO) to analyze periapical bone resorption as we did previously [22]. Scanning was performed at 70 kV and 200 mA with 300 ms integration time. Images at a resolution of 10 μm were acquired and then analyzed. The mandibles were analyzed with a SCANCO Medical Evaluation and SCANCO Medical Visualizer.

### Immunochemistry and TRAP staining

The mandibles were decalcified by 10% EDTA for 30 days. The decalcified samples were embedded in paraffin and sectioned at 10 μm. Tartrate-resistant acid phosphatase (TRAP) staining was carried out using the Acid Phosphatase Leukocyte kit (Sigma, St. Louis, MO). Osteoclasts were defined as multinucleated (≥2) TRAP+ cells on the surface of alveolar bone. The immunohistochemical (IHC) staining was conducted with rabbit anti-TNF-α (GB11188, Servicebio, CN) and anti-IL1β (GB11113, Servicebio) antibodies. Image J was used to quantify and score the average optical density (AOD) of IHC images for inter-group comparison [23].

### Quantification of bacterial load

To compare the bacterial load of *P. gingivalis, Treponema denticola*, and *Fusobacterium nucleatum* between groups, five sections of each decalcified sample were pooled. DNA was isolated using the QIAamp DNA FFPE Tissue kit (Qiagen, Valencia, CA) according to the manufacturer’s instructions. Quantitative amplification was performed by using Bio-Rad iTaq Universal SYBR Green Supermix and Bio-Rad CFX96 system (Bio-Rad Laboratories, Inc., CA). The primers for qPCR were listed in Table S1. The relative abundance of each bacteria was calculated with the 2^−ΔCT^ method, and 16S rRNA was used as internal control.

### Spleen single cell suspension preparation and multicolour flow cytometry

Spleens were removed, homogenized, and centrifuged (350 rpm, 10 min, room temperature). The supernatant was discarded, and red blood cell lysis (BD Biosciences, NJ) was carried out. The cells were re-suspended, and the concentration of the single-cell suspension was adjusted to 1 × 10^7^ cells/mL. A total of 15 immune cells and six cytokines listed in Figure 1(b) were analyzed using multicolor flow cytometry. The single-cell suspensions were stained with antibodies in Table 1. The gating information and calculation methods for cells and cytokines (CD4 + T cell produced IL10, IL17, IL 22, interferon γ (IFNγ), as well as ILC3 produced IL17 and IL 22) were listed in Table S1.

**Table 1.** Antibody used for multicolour flow cytometry.

Antibody	Cat No.	Vendor
Anti-mouse CD16/32	Cat#553141	BD
Anti-mouse CD45 Brilliant Violet 605	Cat#109841	BIOLEND
Anti-mouse CD11c PE Cy7	Cat#117318	BIOLEND
Anti-mouse/humanCD11bPercp Cy5.5	Cat#101228	BIOLEND
Anti-mouse Ly6c FITC	Cat#128006	BIOLEND
Anti-mouse F4/80 Alexa 700	Cat#123130	BIOLEND
Anti-mouse CD137(PDCA-1) Alexa Fluor 647	Cat#127106	BIOLEND
Anti-mouse CD103 PE	Cat#121406	BIOLEND
Anti-mouse CD19 APC Cy7	Cat#115530	BIOLEND
Anti-mouse CD45 Pacific blue	Cat#103126	BIOLEND
Anti-mouse CD4 FITC	Cat#100406	BIOLEND
Anti-mouse CD8a Alexa 700	Cat#100730	BIOLEND
Anti-mouse TCRβchain PE Cy7	Cat#109222	BIOLEND
Anti-mouse TCRγδ Percp Cy5.5	Cat#118118	BIOLEND
Anti-mouse Foxp3 APC	Cat#17-5773-82	AFFYMETRIX/EBIOSCIENCE
Anti-mouse ROR gamma(t) PE	Cat#12-6988-80	AFFYMETRIX/EBIOSCIENCE
APC anti-mouse IL-17A	Cat#506916	BIOLEND
FITC anti-mouse IFN-γ	Cat#505806	BIOLEND
Pacific Blue™ anti-mouse IL-10	Cat#505020	BIOLEND
PE anti-mouse IL-22	Cat# 516404	BIOLEND

The innate immune cells were identified by their surface antigens. For surface antigen detection, cells were fixed in 1% formalin diluted in DMEM overnight, and then, 1 μL FC antibody (anti-mouse CD16/32) was added into 100 μL spleen cell suspension and incubated on ice for 15 min. The antibodies for CD45, CD19, Ly6c, PDCA-1, CD11c, CD11b, F4/80 and CD103 were mixed at concentrations recommended by the manufactures, added into the mixture and incubated in room temperature away from light for 30 min. To detect the immune cells involved in adaptive immune response, immediately after FC and mixed surface antibody incubation (including anti-CD45, CD19, TCRβ, TCRγδ, CD4, and CD8), cells were fixed in 2 mL Fix/Perm buffer (BD Biosciences) for 50 min, permeabilized in permeabilization buffer (BD Biosciences) and centrifuged in room temperature for 10 min. The fixation, permeabilization and centrifugation were done twice. The pellet was resuspended in 100 μL buffer. Mixed antibodies for Foxp3 and Rorγ were added to the cell suspension and incubated at room temperature for 40 min. For cytokine production analysis, the spleen cell suspension was cultured with GolgiStop (BD Biosciences) for 4 h and incubated with antibody mixture (including anti-CD45, CD4, TCRβ, TCRγδ, IL 17a, IFN-γ, IL 22 and IL 10) as described above. The analysis was performed with the KALUZA software, and the proportions of targeted immune cell subsets were calculated first. To prepare the heat maps, the log2 fold change value of each cell/cytokine compared to the HSDM group was calculated for individual PPDM mice. Then, these values of each cell subset were normalized to a scope of [−1.5,1.5], using the formula: relative change = X/(Xmax − Xmin). A heat map was created using the R project.

### Statistical analyses

Group comparisons were performed by the one-way analysis of variance test (ANOVA) followed by Tukey’s test to compare differences between groups, and two-group comparison was performed by the Student’s *t*-test (SPSS v.10.0; SPSS Inc., Chicago, IL). *p* < 0.05 was considered statistically significant.

## Results

**Figure 2.**
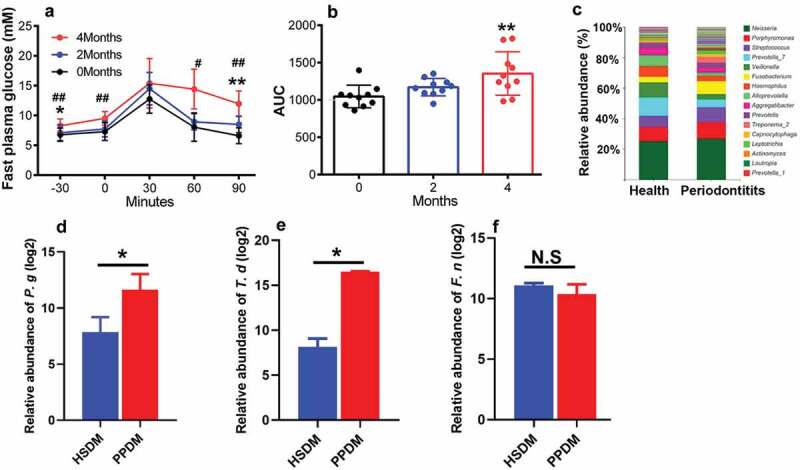
Diabetic mouse model establishment and oral microbiota transplantation. (a) Fast plasma glucose measurement by OGTT at start point, 2 and 4 months; (b) AUC analysis of data present in A; (c) Composition of the salivary microbiota from healthy donates and periodontal patients at genus level. Taxa with relative abundance higher than 1% are present. (d–f) The relative abundances of *P. gingivalis* (*P. g), T. denticola* (*T. d*), and *F. nucleatum* (*F. n*) in periodontitis patient donated microbiome recipient mice (PPDM) and healthy subject donated microbiome recipient mice (HSDM). # (p < 0.05) and ## (p < 0.01) indicate statistically significant difference between 0 and 2 months in A. * (p < 0.05) and ** (p < 0.01) indicate statistically significant difference between 0 and 4 months in A and B; * indicates p < 0.05 in D-F; N.S: not significant. N = 3 for each group.

To investigate the effect of human periodontitis-associated salivary microbiome on the immune responses of DM mice, the high fat food was used to establish an *in vivo* DM animal model. After feeding with high fat food for 4 months, the weight of the mice increased by 32.7 ± 0.46%. Importantly, the fast plasma glucose concentration increased to 8.29 ± 1.12 mM, and the glucose tolerance was significantly impaired compared to the start point (Figure 2(a-b)). These diabetic mice were randomly grouped into HSDM (healthy subject donated microbiome recipient mice) and PPDM (periodontitis patient donated microbiome recipient mice) for OMT and downstream analysis.

The microbiome transplantation was done at the eighth month. Before transplantation, differences within the human salivary microbiome were detected between healthy and periodontal donors by using 16S rRNA sequencing (Figure 2(c)). The genera *Porphyromonas, Streptococcus, Fusobacterium* and *Treponema* had higher relative abundance in the saliva of periodontal patients, while the proportions of the genera *Prevotella_*7, *Veillonella*, and *Haemophilus* were higher in the healthy saliva microbiome.

Six weeks after transplantation, no statistically significant difference was detected between PPDM and HSDM in the levels of ALT, HDL-C, LDL-C, TC, TG, CREA and urea (Table 2). To test if the oral microbial composition of mice in HSDM and PPDM was affected by transplantation, three periodontal pathogens (i.e. *P. gingivalis, T. denticola* and *F. nucleatum*) were selected and their distributions were compared. Elevated relative abundance of *P. gingivalis* (Figure 2(d)) and *T. denticola* (Figure 2(e)) were observed in the PPDM recipient mice, while the difference in the relative abundance of *F. nucleatum* (Figure 2(f)) between groups was not significant.

**Table 2.** Effect of periodontal microbiota on liver and kidney function.

Group	ALT (U/L)	HDL-C (mmol/L)	LDL-C (mmol/L)	TC (mmol/L)	TG (mmol/L)	CREA (umol/L)	UREA (mmol/L)
HSDM	52 ± 5.0	1.3 ± 0.2	0.4 ± 0.1	2.1 ± 0.4	0.6 ± 0.1	4.8 ± 3.5	9.6 ± 2.6
PPDM	51. ± 5.4	1.6 ± 0.3	0.4 ± 0.1	2.4 ± 0.5	0.8 ± 0.1	2.1 ± 0.5	8.5 ± 1.3

HSDM: healthy subject-donated microbiome recipient mice; PPDM: periodontitis patient-donated microbiome recipient mice.

**Figure 3.**
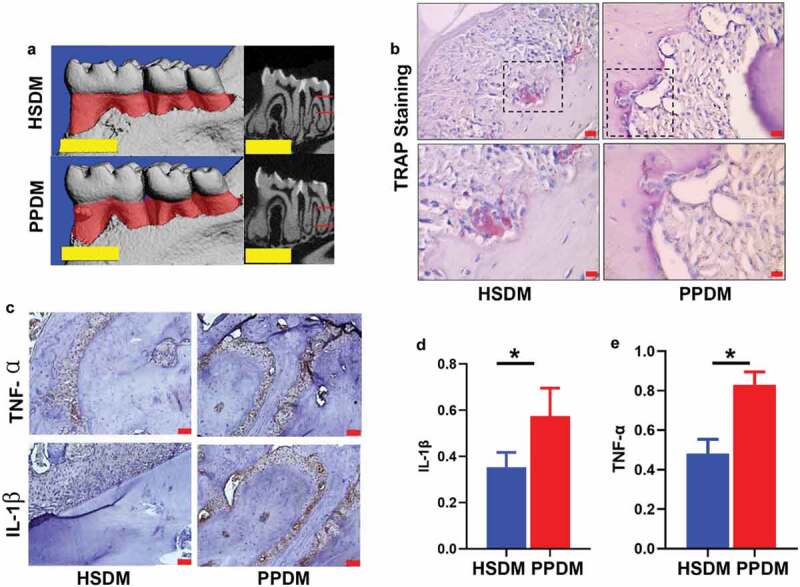
Oral manifestation in recipient mice after human oral microbiome transplantation. (a) microCT images of periodontal tissues at endpoint; scale bar: 1 mm; (b) TRAP staining. Boxes in upper panel are shown magnified in lower panel; scale bar: 50 μm. (c) IHC staining of TNF-α (upper panel) and IL-1β (lower panel); scale bar: 50 μm. AOD quantification of TNF-α (d) and IL-1β (e) in IHC images. HSDM: healthy subject donated microbiome recipient mice; PPDM: periodontitis patient donated microbiome recipient mice; *(p < 0.05) indicates statistically significant difference between groups. N.S: not significant. N = 3 for each group.

The micro-CT scanning (Figure 3a) showed that periodontal lesions between PPDM and HSDM were comparable. However, TRAP+ osteoclasts (Figure 3(b)) and the expression levels of TNF-α and IL-1β (Figure 3(c-e)) within the periodontal tissues were significantly increased in the PPDM group compared to the HSDM group.

**Figure 4.**
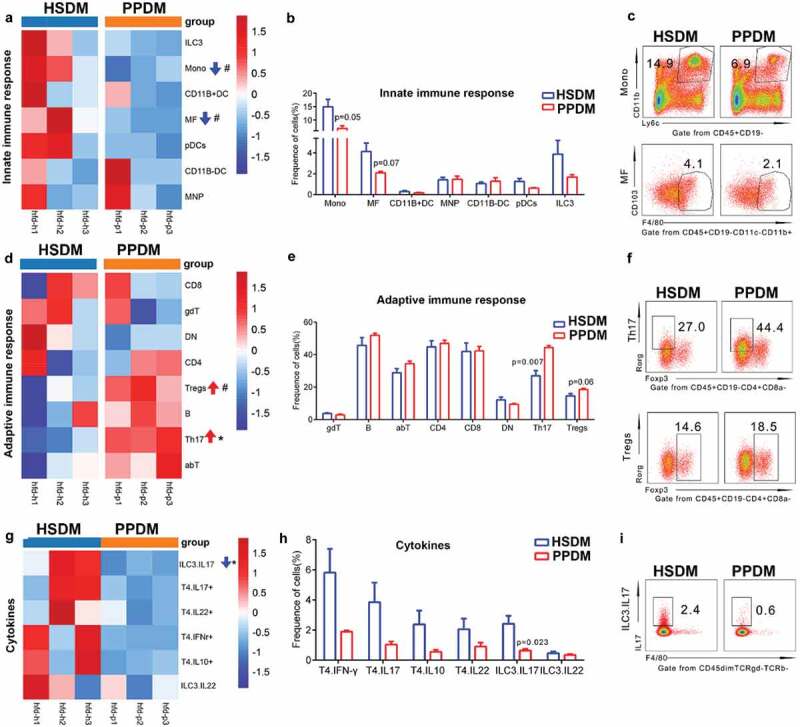
Comparison of innate (a–c), adaptive immune cells (d–f) and cytokine production (g–i) between groups at endpoint. Each column represents one sample. a, d, g: Heatmap of average fold change for cells listed in the right of each column; b, e, h: frequency comparison between groups; c, f, i: scatter plot of cells with significantly different distribution. HSDM: healthy subject donated microbiome recipient mice; PPDM: periodontitis patient donated microbiome recipient mice; DC: dendritic cell; pDCs: plasmacytoid dendritic cells; Mono: monocyte; MNP: mononuclear phagocyte; MF: macrophage; ILC: innate lymphocyte cell; ILC3: innate lymphocyte cell type 3; abT: TCRαβ T cell; gdT: TCRγδ T cell; B: B cell; CD4+: CD4+ cell; CD8+: CD8+ cell; DN: CD4-CD8- T cell; Th17: Th17 T cell; Treg: Foxp3+ regulatory T cell. T4.IFNγ: CD4 + T cell produced IFNγ; T4.IL10+: CD4 + T cell produced IL10; T4.IL17+: CD4 + T cell produced IL17; T4.IL22: CD4 + T cell produced IL22; ILC3. IL17: ILC3 produced IL17; ILC3. IL22: IL3 produced IL22. # indicates p < 0.1 and * indicates p < 0.05. N = 3 for each group.

Multicolor flow cytometry assay was then used to detect the influence of the human oral microbiome on 15 splenic immune cells of DM mice (including seven innate immune cells and eight adaptive immune cells). As shown in Figure 4, there were two immune cells showing different distribution patterns between PPDM and HSDM. Specifically, the proportion of an innate immunocyte, that is, the monocyte, was approximately two times lower in PPDM compared to HSDM (Figure 4 (a~c), p = 0.05). However, the adaptive immunocyte Th17 was up-regulated in PPDM (44.4 ± 1.7% vs 27.0 ± 4.5%, p = 0.007, Figure 4 (d~f)). In addition, the Th17/Tregs ratio increased in the PPDM group compared to HSDM (Figure 4(f)). In summary, the above data showed that the spleen of the PPDM had decreased levels of innate immune cells and increased adaptive immunocytes compared to HSDM. We also tested whether the human periodontitis-associated salivary microbiome can affect the circulating cytokine secretion of splenic immune cells. Interestingly, although the level of ILC3 cells was comparable between groups (Figure 4(b)), the IL17 production of ILC3 cells in PPDM was significantly lower compared to HSDM (2.4 ± 0.7% vs 0.6 ± 0.2%, p < 0.05). Briefly, these data suggested that the dysbiosis of the oral microbiota impaired cytokine production of immune cells in DM mice.

## Discussion

Both diabetes and periodontitis are widespread chronic diseases affecting humans. The bilateral relationship between diabetes and periodontitis has been well accepted although the underlying mechanism has not been well unveiled. Diabetes and periodontitis are thought to share a common pathogenesis such as uncontrolled inflammatory response at both the local and systemic levels. It is generally believed that the oral microbiome plays an important role in regulating the immune response which triggers and exacerbates diabetes. In the present study, we explored the influence of the dysbiotic salivary microbiome from periodontal patients on the immune response of DM mice. By doing so, we aimed to provide clues to understanding immune cells which have potential roles in mediating both diseases.

We established an *in vivo* diabetes mouse model. Quadruple antibiotic treatment was used to normalize the mouse oral microbiome between groups at the baseline and to facilitate the following microbiome transplantation. Spleens have been used as an *in vivo* systemic immune barometer [21,24], thus we sampled splenic cells to analyze the changes of systemic immune response. The capability of the human oral microbiota colonizing germ-free mice has been documented before [25]. Nevertheless, it is important to check the successful colonization of donor’s microbiota on recipient mice in microbiome transplantation-based experiments. However, due to the high similarity in the genome of human- and mouse-derived bacteria, it is quite tough to tell in recipients which strains are from donors and which are not, especially in studies aiming to transplant a complex microbial community containing hundreds of different bacteria. It might be the main reason preventing publications from providing validation data about successful colonization (even in these released very recently) [13,26–28]. As a robust therapeutic and research method, there might be a good strategy to solve this problem very soon. Despite this, we did observe elevated levels of *P. gingivalis* and *T. denticola* in mice receiving a periodontitis patient donated microbiome compared to these transplanted with a healthy subject donated microbiome. These data suggested that the oral microbiota of DM mouse in different groups is changed by transplantation of distinct microbial communities. In parallel, we detected elevated levels of TRAP+ osteoclasts, TNF-α and IL-1β in mice receiving a periodontitis patient donated microbiome. Previous studies revealed that invasion of periodontal pathogens such as *P. gingivalis* enhances the TRAP+ osteoclast pool [29] and induces the IL-1β, TNF-α and IL-6 production [30]. Therefore, the changes of the oral microbiome, TRAP+ osteoclasts, TNF-α and IL-1β in PPDM are consistent.

We noticed that at the endpoint (i.e. 6 weeks after the oral microbiota transplantation), no statistically significant difference was observed between groups in terms of the levels of HDL-C, LDL-C, TC, TG, ATG, CREA, and urea, as well as in the severity of periodontal lesions. These data indicated that after microbiome transplantation, the local and systemic conditions between groups were comparable. Importantly still, these comparable data excluded the possibility that the difference we observed in the immune response of the spleen was not caused by a distinct microbiome but was secondary to the local or systemic changes. The potential reason for the comparable liver and kidney function test results between PPDM and HSDM might be that, oral microbiota transplantation has a mild effect on the liver and kidney function. Considering the elevated TRAP+ osteoclasts and increased levels of periodontitis associated cytokines (e.g. TNF-α and IL-1β) in the PPDM group, one explanation for the similar severity of periodontal lesions between groups may be that the mice were euthanized 6 weeks after transplantation, and it might take more than 6 weeks for significant differences to appear.

Immune cells including innate and adaptive immune cells are crucial components of immune systems. Innate immune cells (i.e. monocytes, macrophages and DC cells) can recognize pathogen-associated molecular patterns (PAMPs) via their pattern-recognition receptors (PRRs), resulting in targeted and specific destruction of the activating organisms by releasing cytotoxic agents or phagocytosis [31]. In this study, we found that the innate immune response in mice receiving a periodontal microbiome was down-regulated, as both monocytes and macrophages had lower relative abundance in these mice. During postnatal life, monocytes can replace resident macrophages in all major organs and adopt their tissue-specific gene expression [32]. Therefore, the changes of monocytes and macrophages in the present study were consistent. Some studies detected increased monocytes/macrophage levels in the peripheral blood and gingiva of periodontitis patients [33] and in the pancreas sections of DM patients and animal models [34]. Since these studies were carried out in populations with either periodontitis or diabetes, the difference between these findings and our observation suggested that co-presence of periodontitis and DM might cause a set of immune cascades different from these we discerned from periodontitis or DM.

We observed elevated proportions of Th17 cells in the PPDM group compared to the HSDM group. Th17 cells are key mediators of alveolar bone resorption during the progression of periodontitis [35]. The level of Th17 cells is positively correlated with the severity of periodontitis, and the proportion of Th17 cells in type 2 diabetic patients was also up-regulated [36–38]. Meanwhile, an up-regulation of the Th17/Tregs ratio was detected. In recent years, the alterations in and the roles of the Th17/Treg balance in type 2 diabetes mellitus has attracted attention, and the Th17/Treg balance is crucial for preventing excessive immune activation, autoimmune responses, and metabolic syndrome pathogenesis [39]. It has been shown that Th17/Treg is a bridge linking the gut microbiota to type 2 diabetes mellitus [40]. Here, in the present study, we found a Th17/Tregs imbalance in mice receiving periodontitis-associated salivary microbiomes. It will be interesting to test if and how the Th17/Treg balance links the oral microbiota to type 2 diabetes mellitus in the future.

Interestingly, Almubarak et al. compared the transcriptome of monocytes/macrophages in gingival tissue of periodontal patients with and without diabetes, and found a significant disruption of monocyte and macrophage homeostasis toward a proinflammatory phenotype [41]. The imbalance within the monocyte-macrophage system was also observed in diabetic patients [42]. These results suggest that both the quantity and function of immunocytes might be affected by the periodontal microbiome in periodontitis patients also suffering with diabetes. Therefore, we set up an experiment to test in our animal model if a dysbiotic oral microbiota will pose an impact on the circulating cytokine production of immune cells. As expected, we found that the ILC3 produced IL17 had a lower level in PPDM mice. There are three subsets of ILCs, and the presence of ILC3 in biopsies of periodontal patients has been reported previously [43]. However, there are no reports in the current literature describing the specific roles of ILC3 produced IL17 in periodontitis and/or DM. Further studies about the roles of ILC3 produced IL17 on the microbiome–periodontitis–diabetes axis are warranted.

Although immune responses in both the oral cavity and distal spleen were changed by a disturbed oral microbiome, the oral cavity and spleen showed site-specific immune activity. Specifically, oral microbiome imbalance increased the levels of osteoclasts, TNF-α and IL-1β in the local gingival tissues, while it decreased the levels of monocytes and ILC3 produced IL17 in the spleen but enriched Th17 cells. These site-specific immune activities indicate how the immune system is fine-tuned by the oral microbiome.

## Conclusion

Within the limitations of this study, the work suggested that a disturbed human oral microbiome induces an immune response in the spleen of diabetic mice, which is characterized by decreased innate immune cells and elevated adaptive immunocytes. Monocytes and Th17 cells might be the key immunocytes for the oral microbiome to regulate the immunity of DM, and oral microbes might also interfere with cytokine production of splenic immune cells.
